# Fermentation-Derived 6-Shogaol from *Zingiber officinale* Rhizome Extract Inhibits Periodontal Biofilm Formation via Modulation of Quorum Sensing-Related Gene Expression

**DOI:** 10.3390/ijms27136013

**Published:** 2026-07-04

**Authors:** Aimin Li, Masafumi Noda, Ikue Hayashi, Narandalai Danshiitsoodol, Masanori Sugiyama

**Affiliations:** Department of Probiotic Science for Preventive Medicine, Graduate School of Biomedical and Health Sciences, Hiroshima University, Kasumi 1-2-3, Minami-ku, Hiroshima 734-8551, Japan; d242910@hiroshima-u.ac.jp (A.L.); bel@hiroshima-u.ac.jp (M.N.); ikue@hiroshima-u.ac.jp (I.H.); naraa@hiroshima-u.ac.jp (N.D.)

**Keywords:** lactic acid bacteria, periodontal pathogens, biofilm, *Zingiber officinale*, 6-shogaol, quorum sensing

## Abstract

Microbial fermentation of plant-derived materials is increasingly recognized as a strategy to enhance biological activity through phytochemical bioconversion. In this study, we investigated the antibiofilm effects of fermented *Zingiber officinale* rhizome extract against major periodontal pathogens and examined the underlying mechanisms. Ginger extract fermented with plant-derived lactic acid bacteria showed significantly greater inhibition of biofilm formation by *Porphyromonas gingivalis*, *Fusobacterium nucleatum*, and *Aggregatibacter actinomycetemcomitans* than non-fermented extract. The inhibitory activity increased with fermentation time, resulting in approximately 60–70% reduction in biofilm formation at higher concentrations. Chromatographic analysis revealed decreased 6-gingerol and increased 6-shogaol levels after fermentation, suggesting bioconversion of 6-gingerol to 6-shogaol. Direct treatment with 6-shogaol inhibited biofilm formation in a dose-dependent manner in all tested pathogens. Quantitative PCR analysis further showed that 6-shogaol significantly downregulated the quorum sensing-related gene *luxS* and multiple adhesion- and virulence-associated genes, including *flp*, *fimA*, *mfa1*, *radD*, *fadA*, *ltxA*, and *rgpB*. These findings indicate that lactic acid bacterial fermentation enhances the antibiofilm activity of ginger extract through increased 6-shogaol production, highlighting its potential as a natural anti-biofilm and anti-virulence agent for periodontal disease prevention and management.

## 1. Introduction

Periodontitis is a complex and common oral disease, which is usually caused by various pathogens in the human mouth through complex synergistic action [1]. The periodontal-related bacteria, *Porphyromonas gingivalis*, *Fusobacterium nucleatum*, and *Aggregatibacter actinomycetemcomitans* play important roles in the bacterial communities that cause periodontitis [2,3,4].

*Aggregatibacter actinomycetemcomitans* is a key pathogen associated with aggressive periodontitis, contributing significantly to biofilm formation and periodontal tissue destruction. It adheres to dental and epithelial surfaces via fimbriae and outer membrane proteins, facilitating colonization and co-aggregation with other oral bacteria such as *F. nucleatum* and *P. gingivalis* [5,6,7]. The bacterium produces potent virulence factors, including leukotoxin and cytolethal distending toxin, which promote host immune evasion and inflammation [8,9]. Through these mechanisms, *A. actinomycetemcomitans* enhances the pathogenic synergy and stability of multispecies periodontal biofilms, thereby exacerbating disease progression.

*Fusobacterium nucleatum* is a Gram-negative anaerobe that serves as a key bridge organism in periodontal biofilm development and disease progression. Its strong adhesion and co-aggregation abilities enable interactions with both early and late colonizers, promoting multispecies biofilm formation [10]. *Fusobacterium nucleatum* modulates host immune responses, contributing to chronic inflammation and immune evasion [11]. By facilitating the incorporation of pathogenic species such as *P. gingivalis* and *A. actinomycetemcomitans*, it drives dysbiosis and periodontal tissue destruction. Moreover, its capacity to disseminate beyond the oral cavity links it to systemic diseases, including colorectal cancer and adverse pregnancy outcomes [12,13,14].

*Porphyromonas gingivalis* is a keystone pathogen in chronic periodontitis, exerting profound effects on the composition and pathogenic potential of the oral biofilm. It adheres to and invades periodontal tissues through fimbriae and adhesion molecules, facilitating immune evasion and chronic infection [15]. *Porphyromonas gingivalis* modulates host responses by altering cytokine production and impairing neutrophil function, promoting inflammation and dysbiosis. Its virulence factors, including gingipains, degrade host proteins and reshape the microbial community [16,17]. Through synergistic interactions with other periodontal species, *P. gingivalis* enhances biofilm pathogenicity and contributes to systemic inflammatory diseases [18].

Our research group has focused on the ability of plant extracts and plant-derived lactic acid bacteria (LABs) to cooperatively generate functional bioactive molecules, and we have been conducting continuous studies in this area. Through various plant–LAB combinations, we have identified multiple functional properties. We have previously found that *Lactobacillus reuteri* (reclassified in April 2020 as *Limosilactobacillus reuteri*) BM53-1, a LAB strain isolated from *Actinidia polygama* (silver vine) flowers, produces a substance that inhibits biofilm formation by a cariogenic bacterium *Streptococcus mutans* when cultured in carrot juice [19].

The biofilm formation also plays a crucial role in the progression of periodontal diseases caused by corresponding pathogens. Bacterial biofilms are structured microbial communities embedded in a self-produced matrix of polysaccharides, proteins, and extracellular DNA, which protects cells from antimicrobial agents and host defenses [20]. Their biofilm formation involves sequential stages of attachment, colonization, maturation, and dispersion, enabling persistence and dissemination. Within biofilms, bacteria exhibit altered metabolism and gene expression, leading to antibiotic resistance up to 1000-fold higher than planktonic cells. Biofilms cause chronic infections and industrial biofouling, highlighting the urgent need for novel therapeutic strategies and inhibitors targeting biofilm formation and maintenance [21].

Our group have continued to explore other plant-derived lactic acid bacteria combinations to identify those effective against periodontal pathogens. Consequently, we found that fermentation of hot water extracts of Zingiber Rhizoma (rhizome of *Zingiber officinale*) with the plant-derived LAB generated a compound that significantly inhibited biofilm formation by periodontal bacteria. Purification and structural analysis revealed the compound to be 6-shogaol, which was suggested to be produced via conversion from 6-gingerol during lactic fermentation as a major candidate bioactive compound. We also investigated the inhibitory mechanism of this activity using quantitative reverse transcription (qRT) PCR analysis.

## 2. Results

### 2.1. Comparison of Antibiofilm Activity Among LAB-Fermented Ginger Extracts

**Figure 1:**
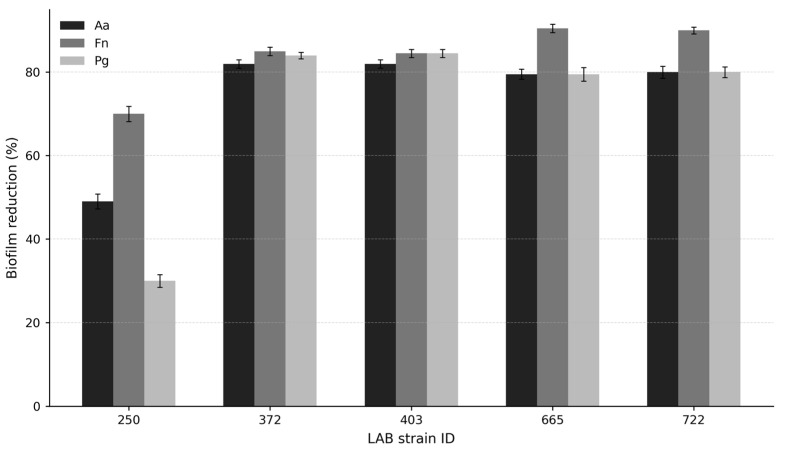
Antibiofilm activity of ginger extracts fermented with different lactic acid bacterial strains against *A. actinomycetemcomitans* (Aa), *F. nucleatum* (Fn), and *P. gingivalis* (Pg). Data are presented as mean ± S.D.

To identify effective lactic acid bacterial strains for the fermentation of ginger extract, the antibiofilm activities of fermented products prepared with different LAB strains were compared against major periodontal pathogens. As shown in Figure 1, the inhibitory effects varied among strains. Among the tested about 750 strains, strains no. 372, 403, 665, and 722 (previously identified as *Enterococcus faecalis*, *Leuconostoc pseudomesenteroides*, *Lactiplantibacillus plantarum*, and *L. plantarum*, respectively) exhibited strong antibiofilm activity against *A. actinomycetemcomitans*, *F. nucleatum*, and *P. gingivalis*, with strains no. 665 and 722 showing the highest overall activity. In contrast, strain 250 (previously identified as *Enterococcus* sp.) showed relatively weaker inhibition, especially against *P. gingivalis*. These results indicate that the antibiofilm activity of fermented ginger extract is strongly influenced by the LAB strain used for fermentation.

**Figure 2:**
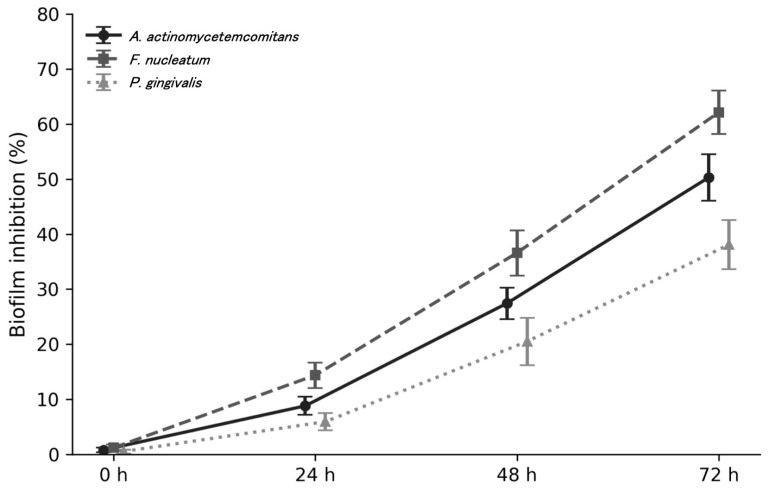
Effect of fermentation time on the antibiofilm activity of fermented ginger extract prepared with strain no. 722 against periodontal pathogens. Biofilm inhibition increased with fermentation time. Data are presented as mean ± S.D.

To determine the optimal fermentation period, the antibiofilm activity of ginger extract fermented for different durations was evaluated. As shown in Figure 2, non-fermented ginger extract showed little or no antibiofilm activity under the tested conditions, whereas biofilm inhibition increased progressively with fermentation time in all three tested pathogens. Compared with the non-fermented control (0 h), the inhibitory activity became evident after 24 h of fermentation, increased further at 48 h, and reached the highest level at 72 h. Among the tested pathogens, *F. nucleatum* was the most sensitive, whereas *P. gingivalis* showed relatively lower inhibition. These findings suggest that prolonged fermentation promotes the generation or accumulation of active antibiofilm compounds.

### 2.2. Confirmation of Antibiofilm Substance Obtained from the Fermented Ginger Extract

**Figure 3:**
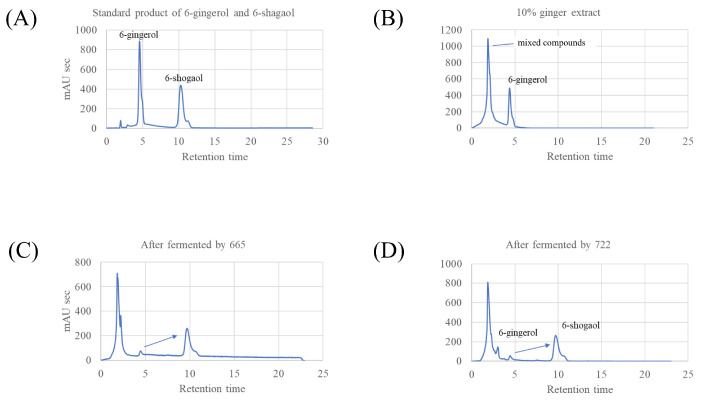
HPLC chromatograms of ginger-related samples. As follows: (**A**) Standard mixture of 6-gingerol and 6-shogaol. (**B**) Unfermented 10% ginger extract. (**C**) Ginger extract fermented with LAB strain 665. (**D**) Ginger extract fermented with LAB strain 722. Fermentation was associated with a decrease in 6-gingerol and the appearance of a 6-shogaol peak. The arrows indicate conversion of 6-gingerol to 6-shogaol during fermentation.

To investigate the chemical changes associated with fermentation, high-performance liquid chromatography (HPLC) chromatograms of standard compounds, unfermented ginger extract, and fermented ginger extract were compared. As shown in Figure 3, the standard mixture exhibited distinct peaks corresponding to 6-gingerol and 6-shogaol. In the unfermented ginger extract, 6-gingerol was clearly detected, whereas the 6-shogaol peak was absent or barely detectable. In contrast, after fermentation with the selected LAB strains, particularly strains no. 665 and 722, the chromatograms showed a marked decrease in the 6-gingerol peak together with the appearance of a new peak corresponding to 6-shogaol. These results suggest that fermentation induced the bioconversion of 6-gingerol into 6-shogaol.

**Figure 4:**
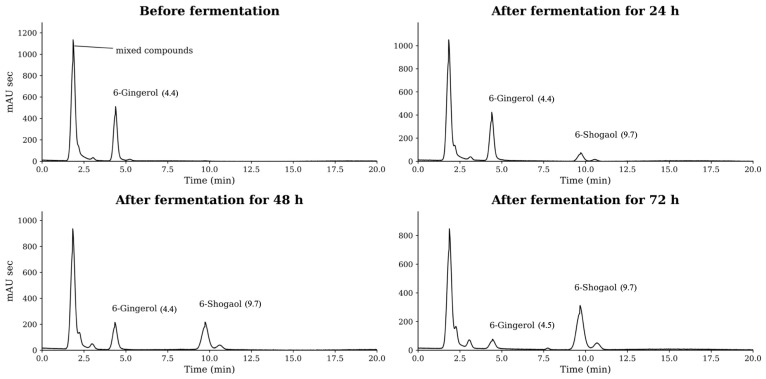
HPLC profiles of fermented ginger extract at 0, 24, 48, and 72 h-fermentation. Numbers in parentheses indicate retention time.

Next, to investigate the time-dependent changes during fermentation, culture supernatants were collected at 24 h intervals following fermentation with strain no. 722, and the relative abundances of 6-gingerol and 6-shogaol were compared by HPLC analysis (Figure 4). As shown in Figure 4, the decrease in 6-gingerol was temporally synchronized with the increase in 6-shogaol, suggesting that fermentation with strain no. 722 is associated with the bioconversion of 6-gingerol into 6-shogaol.

**Figure 5:**
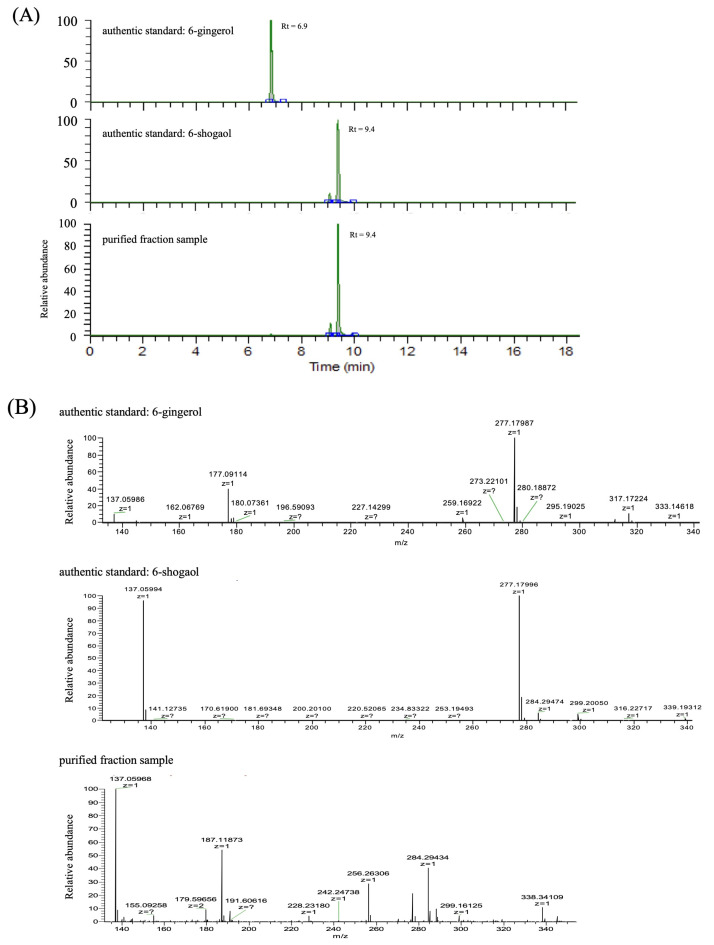
Chromatographic profiles (**A**) and MS/MS fragmentation ions (**B**) of the LC-MS/MS analyses of authentic 6-gingerol standard, authentic 6-shogaol standard, and purified fraction sample collected by preparative HPLC. The flags z = ? refer to an unknown or undetermined charge state.

The purified fraction in 6-shogaol following LAB fermentation by HPLC was subsequently confirmed by LC–MS/MS analysis. Specifically, high-resolution accurate mass (HRAM) LC–MS/MS analyses were performed using authentic standards of 6-gingerol and 6-shogaol on an Orbitrap Eclipse mass spectrometer. Comparison of the chromatographic and mass spectral data confirmed that the isolated fraction corresponded to 6-shogaol (Figure 5). In positive-ion LC–ESI–MS analysis, the authentic standard of 6-gingerol (C_17_H_26_O_4_) produced ions corresponding to [M+H]^+^, [M−H_2_O+H]^+^, [M+Na]^+^, and [M+K]^+^ at *m*/*z* 295.3, 277.2, 317.2, and 333.2, respectively. The authentic standard of 6-shogaol (C_17_H_24_O_3_) generated [M+H]^+^, [M+Na]^+^, and [M+K]^+^ ions at *m*/*z* 277.2, 299.2, and 315.1, respectively. Because 6-gingerol contains a hydroxyl group in its side chain, the dehydrated ion [M−H_2_O+H]^+^ was observed as the predominant ion, whereas the protonated molecular ion [M+H]^+^ was relatively weak. In contrast, 6-shogaol possesses a carbonyl group rather than a hydroxyl group in the side chain, and therefore no dehydrated ion ([M−H_2_O+H]^+^) was detected. MS/MS spectra were acquired using higher-energy collisional dissociation (HCD) at collision energies of 20%, 35%, and 60%. For 6-gingerol, the dehydrated precursor ion [M−H_2_O+H]^+^ (*m*/*z* 277) generated fragment ions at *m*/*z* 177, 162, 145, and 117. Among these, the most intense fragment ion, *m*/*z* 177.0910, was selected as the characteristic qualitative ion of 6-gingerol. For 6-shogaol, the protonated precursor ion [M+H]^+^ (*m*/*z* 277) generated fragment ions at *m*/*z* 137, 122, and 94, with *m*/*z* 137.0597 being the most abundant and therefore selected as the characteristic qualitative ion of 6-shogaol.

LC–MS/MS analysis of the HPLC-isolated fraction obtained from LAB fermentation revealed a fragment ion peak at *m*/*z* 137 derived from the precursor ion at *m*/*z* 277, eluting at the same retention time as the authentic 6-shogaol standard. LC–MS/MS analysis using the high-resolution Orbitrap mass spectrometer detected precursor and fragment ions identical to those of the authentic 6-gingerol and 6-shogaol standards. In all cases, the measured masses were within 5 ppm of their theoretical values. Compound identification was based on the agreement of both chromatographic retention times and mass spectra with those of the authentic standards, specifically Rt = 6.9 min for 6-gingerol and Rt = 9.4 min for 6-shogaol. Therefore, the identity of 6-gingerol and 6-shogaol was confirmed based on three criteria: (i) retention-time matching with authentic reference standards, (ii) high-accuracy mass measurements within 5 ppm of the theoretical masses, and (iii) agreement of characteristic MS/MS fragmentation patterns.

**Figure 6:**
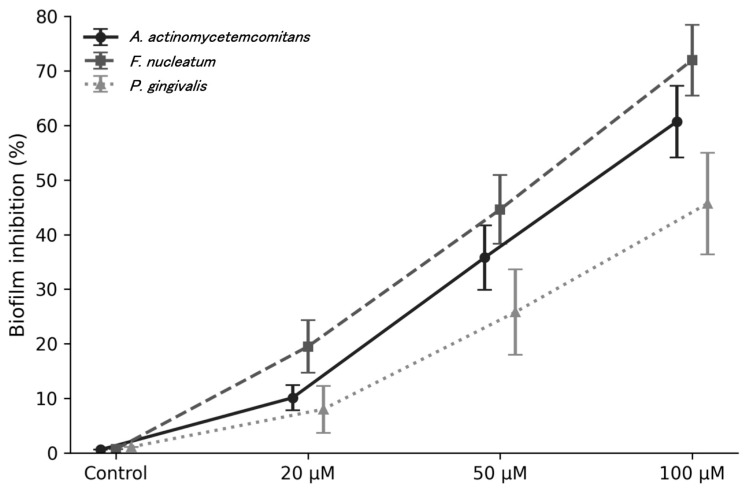
Dose-dependent inhibition of biofilm formation by 6-shogaol in *A. actinomycetemcomitans*, *F. nucleatum*, and *P. gingivalis*. Data are presented as mean ± S.D.

Based on the compositional changes observed after fermentation, the antibiofilm activity of 6-shogaol was evaluated directly. As shown in Figure 6, treatment with 6-shogaol inhibited biofilm formation in a dose-dependent manner in *A. actinomycetemcomitans*, *F. nucleatum*, and *P. gingivalis*. At 20 μM, only modest inhibition was observed, whereas treatment at 50 and 100 μM produced substantially stronger effects. At 100 μM, biofilm inhibition reached approximately 60% in *A. actinomycetemcomitans*, 70% in *F. nucleatum*, and 45% in *P. gingivalis*. Among the tested bacteria, *F. nucleatum* showed the highest sensitivity to 6-shogaol.

**Figure 7:**
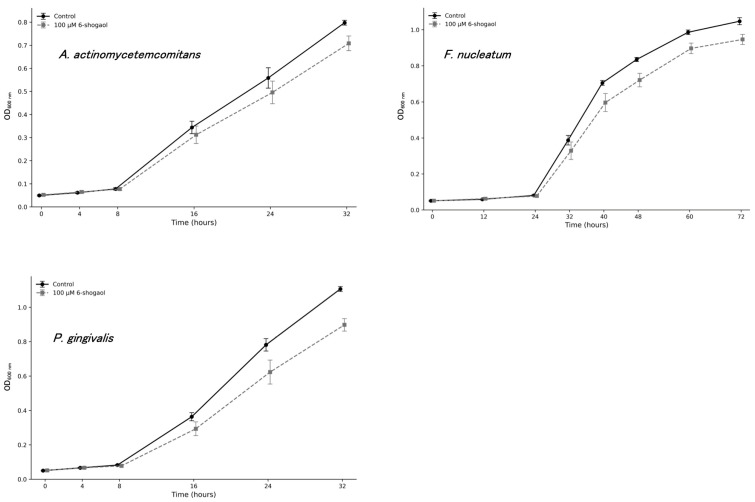
Growth curves of *A. actinomycetemcomitans*, *F. nucleatum*, and *P. gingivalis* cultured in the presence/absence of 100 μM 6-shogaol.

To further evaluate whether 6-shogaol affected bacterial growth, optical density (OD) values at 600 nm were monitored over time in the presence of 100 μM 6-shogaol, a concentration that exhibited potent antibiofilm activity, and compared with those of untreated controls (Figure 7).

In *A. actinomycetemcomitans*, OD values in the 6-shogaol-treated group were comparable to those of the control group during the first 8 h of incubation, indicating no apparent growth inhibition during the early growth phase. At 16 h and thereafter, the OD values in the treated group were approximately 9.2–11.2% lower than those in the control group. However, bacterial growth continued over time in the presence of 6-shogaol, suggesting only a modest reduction in growth rather than strong inhibition. In *P. gingivalis*, OD values were approximately 19% lower in the 6-shogaol-treated group than in the control group between 16 and 32 h of incubation. Although the effect on growth was the greatest among the three tested species, bacterial proliferation was still observed in the presence of 6-shogaol, indicating that complete growth inhibition did not occur. In *F. nucleatum*, OD values in the treated group were reduced by approximately 15.1–13.7% compared with those in the control group between 32 and 48 h of incubation. Thereafter, the difference between the two groups gradually diminished, and the final reduction in OD at 72 h was approximately 9.6%. These findings suggest that 6-shogaol transiently delayed bacterial growth, followed by partial recovery during the later stages of incubation.

Collectively, these results indicate that 6-shogaol did not markedly inhibit the growth of any of the three periodontal pathogens. The impact on bacterial growth was relatively minor in *A. actinomycetemcomitans* and *F. nucleatum*, whereas a somewhat greater effect was observed in *P. gingivalis*. Therefore, although growth suppression may partially contribute to the observed inhibition of biofilm formation, it is unlikely to fully account for the previously demonstrated antibiofilm effects of 6-shogaol. These findings suggest that the inhibitory effects of 6-shogaol on biofilm formation are not solely attributable to growth inhibition or bactericidal activity, but are more likely associated with interference with bacterial adhesion and/or biofilm developmental processes.

### 2.3. Effect of 6-Shogaol on Biofilm- and Virulence-Related Gene Expression in A. actinomycetemcomitans

**Figure 8:**
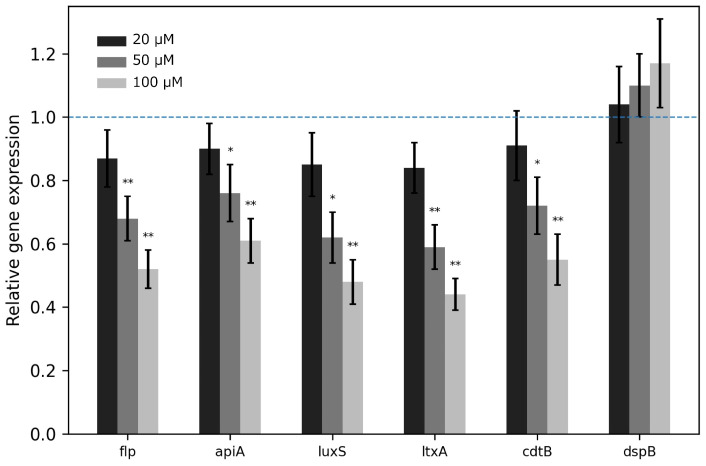
Relative expression of biofilm- and virulence-related genes in *A. actinomycetemcomitans* after treatment with 20, 50, and 100 μM 6-shogaol. Expression levels were normalized to 16S rRNA and are shown relative to the control (blue dashed line). * <0.05, ** <0.01.

To explore the molecular basis underlying the antibiofilm effect of 6-shogaol, the expression of biofilm- and virulence-related genes was analyzed by qPCR. In *A. actinomycetemcomitans*, 6-shogaol treatment downregulated several target genes in a dose-dependent manner (Figure 8). Notably, the quorum sensing-related gene *luxS* was significantly reduced. In addition, the expression of adhesion-related gene *flp* and virulence-associated genes such as *apiA*, *ltxA*, and *cdtB* was also suppressed. By contrast, *dspB* showed relatively limited change compared with the other genes.

### 2.4. Effect of 6-Shogaol on Gene Expression in F. nucleatum

**Figure 9:**
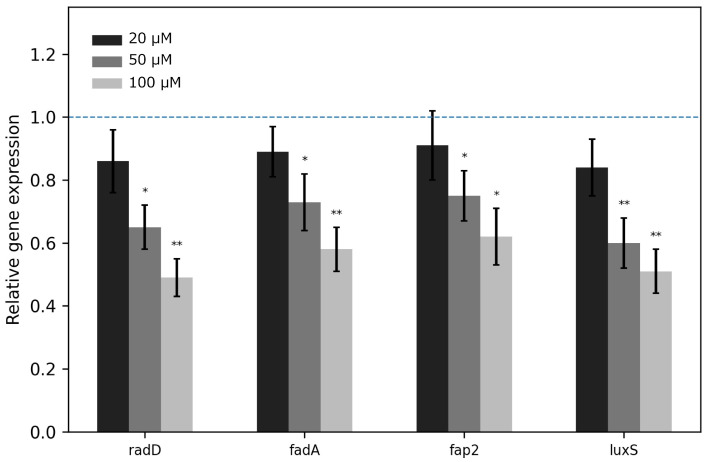
Relative expression of adhesion- and quorum sensing-related genes in *F. nucleatum* after treatment with 20, 50, and 100 μM 6-shogaol. Expression levels were normalized to 16S rRNA and are shown relative to the control (blue dashed line). * <0.05, ** <0.01.

In *F. nucleatum*, 6-shogaol treatment also reduced the expression of genes involved in adhesion and quorum sensing (Figure 9). The transcript levels of *radD*, *fadA*, and *fap2* were decreased, particularly at higher concentrations. The expression of *luxS* was likewise downregulated, suggesting that 6-shogaol may interfere with autoinducer-2 (AI-2)-associated quorum sensing pathways in *F. nucleatum*. These results support the view that the compound suppresses biofilm formation partly through modulation of bacterial adhesion and communication systems.

### 2.5. Effect of 6-Shogaol on Gene Expression in P. gingivalis

**Figure 10:**
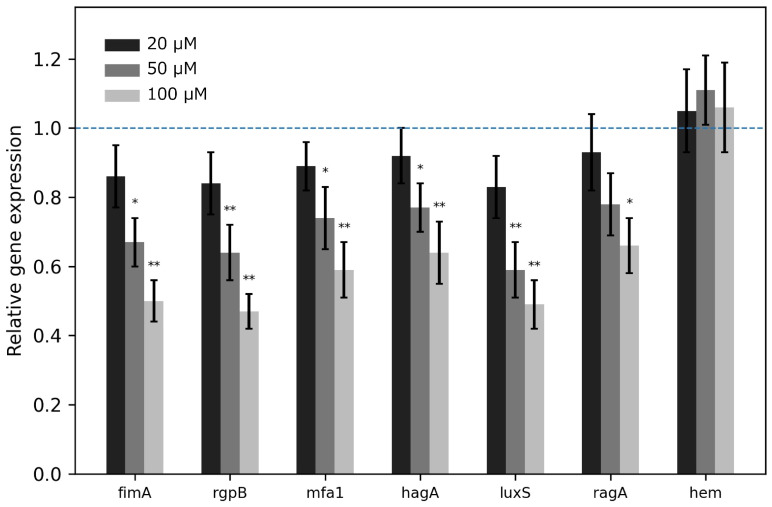
Relative expression of virulence- and quorum sensing-related genes in *P. gingivalis* after treatment with 20, 50, and 100 μM 6-shogaol. Expression levels were normalized to 16S rRNA and are shown relative to the control (blue dashed line). * <0.05, ** <0.01.

Similar effects were observed in *P. gingivalis* (Figure 10). Treatment with 6-shogaol resulted in dose-dependent downregulation of multiple genes associated with adhesion, virulence, and quorum sensing. Specifically, the expression levels of *fimA*, *rgpB*, *mfa1*, *hagA*, *luxS*, and *ragA* were reduced after treatment, whereas hem showed only minor changes. These findings suggest that 6-shogaol broadly affects virulence-associated regulatory pathways in *P. gingivalis*.

## 3. Discussion

Ginger, derived from *Z. officinale*, has long been recognized as a medicinal and functional food material with diverse biological activities, including antioxidant, anti-inflammatory, antimicrobial, anticancer, neuroprotective, cardiovascular protective, anti-obesity, and antidiabetic effects [22,23,24,25,26,27]. Owing to these multifunctional properties, ginger and its bioactive constituents have attracted increasing attention as promising candidates for the development of functional foods and nutraceuticals aimed at the prevention and management of chronic inflammatory diseases, including periodontal disease. In recent years, the importance of natural compounds capable of suppressing bacterial virulence and biofilm formation, rather than simply exerting bactericidal effects, has become increasingly recognized as a novel strategy for controlling oral infectious diseases.

In the present study, fermentation of ginger extract with selected LAB strains significantly enhanced its antibiofilm activity against major periodontal pathogens, including *A. actinomycetemcomitans*, *F. nucleatum*, and *P. gingivalis*. Importantly, the degree of inhibition differed among LAB strains, suggesting that strain-specific metabolic properties strongly influence the production of bioactive metabolites during fermentation. Among the tested strains, no. 665 and 722, exhibited particularly strong antibiofilm activity against all three pathogens. Furthermore, the inhibitory effects increased progressively with fermentation time and reached maximal levels after 72 h of fermentation. These findings suggest that the prolonged fermentation promotes the generation or accumulation of biologically active compounds responsible for the observed antibiofilm effects.

One of the major findings of this study was the identification of 6-shogaol as a key active compound associated with the enhanced antibiofilm activity of fermented ginger extract. HPLC and LC-MS/MS analyses demonstrated that fermentation was accompanied by a marked decrease in 6-gingerol together with the appearance of a distinct 6-shogaol peak, strongly suggesting bioconversion during the fermentation process. Although several previous studies have reported antibiofilm or antibacterial activities of ginger extracts against periodontal pathogens, direct evidence identifying the responsible active compound has remained limited. Sundaram et al. reported that ginger-derived exosome-like nanoparticles inhibit the growth and biofilm formation of *P. gingivalis* and additionally suppress the growth of *A. actinomycetemcomitans* and *F. nucleatum* [28]. Similarly, Jayakumar et al. and Awad et al. demonstrated antibacterial and antibiofilm activities of ginger extracts against *F. nucleatum* and *A. actinomycetemcomitans*, respectively [29,30]. However, the specific ginger-derived constituent directly responsible for these inhibitory effects had not been clearly identified. Our results demonstrated, for the first time, that 6-shogaol directly suppresses biofilm formation in not only *P. gingivalis* and *A. actinomycetemcomitans*, but also *F. nucleatum*.

This finding is particularly important because these three bacterial species play distinct yet cooperative roles during periodontal disease progression. *Aggregatibacter actinomycetemcomitans* and *P. gingivalis* contribute directly to tissue destruction and immune dysregulation through the production of various virulence factors and toxins, whereas *F. nucleatum* functions as a bridging organism that facilitates coaggregation between early and late colonizers during polymicrobial biofilm maturation [31,32,33,34]. Therefore, the ability of 6-shogaol to inhibit biofilm formation across multiple periodontal pathogens suggests that this compound may exert broad-spectrum effects against complex periodontal biofilm communities rather than acting on a single bacterial species alone.

The compound 6-shogaol is a pungent phenolic compound naturally generated from the dehydration of 6-gingerol [35]. It has been reported that 6-shogaol exhibits stronger biological activities than 6-gingerol, including enhanced antioxidant, anti-inflammatory, and antimicrobial effects. Structurally, the α,β-unsaturated carbonyl group present in 6-shogaol increases its electrophilic reactivity and hydrophobicity, potentially contributing to improved interaction with bacterial membranes and intracellular targets. Although 6-shogaol can be chemically produced under heat- or acid-mediated conditions, reports describing microbial biotransformation of 6-gingerol into 6-shogaol remain limited [36,37]. Recently, Kim et al. demonstrated that fermentation of aqueous ginger extract using two *L. plantarum* strains increased the concentration of 6-shogaol [38]. However, the decrease in 6-gingerol observed in their study did not fully correspond to the increase in 6-shogaol, suggesting that fermentation-induced phytochemical changes may involve more complex metabolic pathways than simple dehydration alone.

Considering that LAB fermentation lowers environmental pH through organic acid production, acidic fermentation conditions may partly contribute to the conversion process observed in the present study. Previous reports by another group have also indicated that 6-gingerol and 6-shogaol can interconvert under acidic conditions [39]. Regarding the potential effect of pH, we previously measured the pH of the fermented samples in preliminary assay and found that it was generally in the range of 3.7–4.0. Bhattarai et al. reported, in a stability study conducted in aqueous solutions, that the degradation of 6-gingerol is dependent on both pH and temperature, and that 6-gingerol was most stable at approximately pH 4 among the conditions tested (pH 1, 4, and 7) [39]. Similarly, Ok and Jeong demonstrated that the residual amount of 6-gingerol was higher at pH 4 than at pH 1 or pH 7 [35]. To examine this possibility, the pH of MRS medium was adjusted to approximately 4, supplemented with a sufficient amount of 6-gingerol, and incubated statically for 3 days prior to HPLC analysis in preliminary assay. No clear conversion of 6-gingerol into 6-shogaol has been observed under these conditions. Therefore, it seems that, at least under pH conditions comparable to those used in the present fermentation experiments, the contribution of spontaneous conversion induced solely by acidic conditions to our experimental results is limited. In addition, microbial enzymes produced by LAB strains may facilitate oxidation–reduction reactions or other secondary metabolic pathways involved in phytochemical transformation. The strain-dependent differences observed in our experiments further support the possibility that specific bacterial metabolic activities contribute to the efficient generation of 6-shogaol. Therefore, the enhanced antibiofilm activity of fermented ginger extract likely reflects not only simple chemical conversion but also broader fermentation-mediated modulation of phytochemical composition.

The direct antibiofilm activity of 6-shogaol observed in this study was accompanied by dose-dependent downregulation of multiple adhesion-, quorum sensing-, and virulence-related genes in all tested bacterial species. Notably, the quorum sensing-associated gene *luxS* was consistently suppressed in three pathogens. The LuxS/AI-2 signaling system plays a central role in bacterial interspecies communication, biofilm maturation, and regulation of virulence-associated behaviors in oral polymicrobial communities [40]. Therefore, suppression of *luxS* expression suggests that 6-shogaol interferes with bacterial communication pathways required for coordinated biofilm development.

Particularly noteworthy was the strong response observed in *F. nucleatum*, which showed the highest sensitivity to 6-shogaol among the tested bacteria. *Fusobacterium nucleatum* acts as a central bridging species within oral biofilms by promoting coaggregation among diverse bacterial species [41]. Therefore, inhibition of adhesion molecules such as *radD*, *fadA*, and *fap2*, together with suppression of *luxS* expression, may disrupt the structural organization and stability of multispecies periodontal biofilms. Similarly, in *P. gingivalis*, 6-shogaol downregulated genes associated with fimbrial adhesion (*fimA* and *mfa1*), proteolytic virulence factors (*rgpB*), hemagglutination (*hagA*), and outer membrane antigen function (*ragA*) [42]. In *A. actinomycetemcomitans*, genes associated with leukotoxin production (*ltxA*), cytolethal distending toxin (*cdtB*), and adhesion (*flp*) were also significantly suppressed [43,44]. Collectively, these findings indicate that 6-shogaol exerts not only antibiofilm effects but also broad antivirulence activities targeting multiple pathogenic mechanisms. More detailed functional assays, such as AI-2 quantification or reporter assays, will be required in future studies to confirm how 6-shogaol regulate quorum sensing systems.

Beyond periodontal pathogens, 6-shogaol has also been reported to inhibit biofilm formation in cariogenic and opportunistic microorganisms such as *Streptococcus mutans* and *Candida* species [45,46]. The proposed mechanisms include disruption of bacterial membrane integrity, increased membrane permeability, and interference with quorum sensing pathways [46]. Our finding is consistent with these previous observations and further extend the potential applicability of 6-shogaol to periodontal disease-associated biofilms. Importantly, suppression of virulence and communication systems without necessarily inducing complete bacterial killing may reduce selective pressure for the emergence of antimicrobial resistance. Such anti-virulence approaches have recently attracted attention as alternative therapeutic strategies against chronic biofilm-associated infections.

Ginger possesses a long history of dietary and medicinal use and is generally regarded as safe for human consumption. Likewise, LAB fermentation is widely utilized in food production and may represent an effective strategy for enhancing the functionality of plant-derived materials. Therefore, the present findings suggest potential relevance for future oral healthcare research, but that additional studies, including cytotoxicity testing using oral cells, polymicrobial biofilm models, and stability evaluation under oral-like conditions, are required before practical applications of fermented ginger extract enriched in 6-shogaol.

Several limitations of this study should be acknowledged. First, all experiments were conducted under in vitro conditions, and the complex ecological environment of the oral cavity could not be fully reproduced. Second, although representative periodontal pathogens were examined individually, actual periodontal biofilms consist of highly diverse multispecies microbial communities interacting dynamically with host immune responses. Therefore, further studies using polymicrobial biofilm models and animal models are necessary to confirm the clinical relevance of the present findings. Third, although 6-shogaol appeared to be a major active compound responsible for the enhanced antibiofilm activity, the involvement of additional fermentation-derived metabolites cannot be excluded. Fermentation was associated with broad phytochemical alterations, and other bioactive compounds may contribute synergistically to the observed effects. Comprehensive metabolomic analyses and detailed mechanistic investigations will therefore be required to clarify the complete bioconversion pathways induced during LAB fermentation.

In conclusion, the present study demonstrates that the fermentation with LAB significantly enhanced the antibiofilm activity of ginger extract against major periodontal pathogens through fermentation-associated phytochemical modification. In particular, the increased production of 6-shogaol was strongly associated with enhanced inhibitory effects on biofilm formation, quorum sensing, adhesion, and virulence-related gene expression. These findings suggest that fermented ginger extract may represent a promising natural antibiofilm and anti-virulence material for the prevention and management of periodontal diseases. Further studies employing minimal inhibitory concentration (MIC) and viability assays will help to clarify the relative contribution of growth inhibition to the antibiofilm activity of 6-shogaol.

## 4. Materials and Methods

### 4.1. Bacterial Strains and Culture Conditions

The bacterial strains used in the present study and their culture conditions are listed in Table 1. To prepare seed culture, LAB strains were cultivated using de Man, Rogosa, and Sharpe (MRS) broth (Merck KGaA, Darmstadt, Germany) as the standing cultivation. The LAB strains used in this study were selected from a “plant-derived LAB library,” which has been continuously established and expanded through ongoing isolation and identification efforts by our research group [47], were subsequently subjected to the following screening assays. Periodontal-related strains were cultured in modified Gifu Anaerobic Medium (GAM) bouillon (Nissui Pharmaceutical Co., Ltd., Tokyo, Japan) under anaerobic conditions using anaerobic jar and pouches (AnaeroPack System, Mitsubishi Gas Chemical Company, Inc., Tokyo, Japan).

### 4.2. Preparation of the Medicinal Herbal Extract

Small, dried pieces of Zingiber Rhizoma (purchased from Kojima Kampo, Osaka, Japan) was suspended in distilled water to final concentration of 10% (*w*/*v*), and heated at 105 °C for 30 min. After cooling, the extract was centrifuged to remove debris, and the resultant supernatant was filtered with a membrane filter (pore size 0.22 μm, Toyo Roshi Kaisha, Ltd., Tokyo, Japan). To obtain the fermented extract, the LAB cells, which were collected from the seed culture and resuspended into phosphate-buffered saline (PBS), were inoculated into the prepared extract (final to 1% (*v*/*v*)) and fermented at 37 °C. After appropriate cultivation time (72 h for initial screening, 24–72 h for main experiment), the cell debris were removed by centrifugation, and the obtained supernatants were membrane-filtered (0.22 μm). Both unfermented and fermented extracts were stored in a 4 °C refrigerator until use.

### 4.3. Screening of Biofilm-Inhibiting Samples

The test samples were supplemented into the modified GAM bouillon (final to 50% (*v*/*v*)) to evaluate whether the biofilm formation of the pathogens was inhibited. Briefly, by using a 96-well cell culture plate (Watson Co, Ltd., Tokyo, Japan), the seed cultures of *A. actinomycetemcomitans*, *P. gingivalis*, and *F. nucleatum* (24, 24, and 36 h cultivation, respectively) were inoculated into 100 μL of the fresh modified GAM bouillon (final to 2% (*v*/*v*)). Subsequently, 100 μL of each test sample was added, and the mixture prepared in the 96-well plate was then incubated statically under anaerobic conditions for 24 h.

After the cultivation period, the amount of formed biofilm was calculated by crystal violet method, in which the amount of absorbed dye correlates with dried biofilm mass [48]. Briefly, the culture medium was carefully removed from each well, followed by gentle washing of the wells three times with distilled water. The cells adhered to the wells were then fixed using methanol for 15 min. After removal of the methanol and air-drying of the plates, the biofilms were stained with 200 μL of 0.1% (*w*/*v*) crystal violet. Excess stain was then removed by distilled water. Finally, the crystal violet bound to the biofilm was dissolved in 200 μL of 33% (*v*/*v*) acetic acid solution, and the reduction in biofilm was determined by measuring the *A*_595_ using a microplate absorbance reader (iMark, Bio-Rad Laboratories, Inc., Hercules, CA, USA).

When necessary, 6-shogaol standard (described later) was supplemented to cell culture medium at concentration of 20, 50, and 100 μM. For preparing the growth curve, a portion of the culture medium was collected during the standing cultivation, and its turbidity at 600 nm was measured using the same plate reader.

### 4.4. Analyses of Active Substances in Fermented Zingiber Rhizoma Extract

The purification of compounds contained in the test samples was performed using a HPLC instrument (JASCO Corporation, Tokyo, Japan) with a Crestpak C18S column (5 μm particle size, 4.6 mm i.d. × 150 mm L, JASCO Corporation) as follows: 55% (*v*/*v*) methanol was used as a mobile phase at a flow rate of 1 mL/min for 30 min. After drying the test samples in vacuo, each residue was dissolved in methanol and membrane-filtered (pore size 0.45 μm). By monitoring the chromatograms at 280 nm, the chromatograms of the samples before and after fermentation were compared with 6-gingerol and 6-shogaol standards (FUJIFILM Wako Pure Chemical Corporation, Osaka, Japan). Each standard was dissolved in 99.8% (*v*/*v*) methanol to final concentration of 1 mg/mL as stock solution. The method of this experiment partly draws on the research methods of predecessors [49,50].

### 4.5. LC-MS/MS Analysis

LC-MS/MS analysis was performed using an Orbitrap Eclipse Tribrid mass spectrometer (Thermo Fisher Scientific K.K., Tokyo, Japan) equipped with an electrospray ionization (ESI) source coupled to a Vanquish UHPLC system (Thermo Fisher Scientific). Separation was achieved on a 2.1 × 100 mm ACQUITY UPLC HSS T3 1.8 μm (Waters Corpration, Milford, MA, USA) using water containing 0.1% formic acid (solvent A) and acetonitrile containing 0.1% formic acid (solvent B) under gradient elution conditions. The mobile phase gradient program was the following: 0 to 2min, 40%B; 2 to 10 min, 90%B; 10 to 13 min, 90%B; 13 to 13.1 min, 40%B; 13.1 to 19 min, 40%B. The flow rate was 0.25 mL/min, and the column temperature was kept at 40 °C.

Mass spectra were acquired in positive-ion mode with a spray voltage of 3.5 kV, ion transfer tube temperature of 350 °C, and Orbitrap resolution of 60,000. Full-scan mass spectra were collected over the range *m*/*z* 100–1000. Data-dependent MS/MS spectra were acquired using HCD fragmentation.

Authentic standards of 6-gingerol and 6-shogaol were analyzed under identical conditions to determine retention times, accurate masses, and MS/MS fragmentation patterns. Compounds detected in fermentation sample were assigned by comparison of retention time, accurate mass (mass error < 5 ppm), and MS/MS spectra with those of authentic standards and or mzCloud database.

### 4.6. RNA Extraction and qRT-PCR Analysis

Total RNA extraction and reverse transcription reaction were performed using a NucleoSpin RNA Plus Kit (Macherey-Nagel GmbH &Co. KG, Düren, Germany) and ReverTra Ace qPCR RT Master Mix with gDNA Remover (Toyobo Co., Ltd., Osaka, Japan), respectively, in accordance with the manufacturer’s instructions. The qRT-PCR was carried out on the CFX Connect Real-Time PCR Detection System (Bio-Rad Laboratories, Inc.) with the KAPA SYBR FAST qPCR Kit Master Mix (2×) Universal (Kapa Biosystems, Woburn, MA, USA). The reaction was conducted as follows: initial denaturation at 95 °C for 30 s, followed by 40 cycles of 5 s at 95 °C and 30 s at 60 °C. The target genes were amplified with the corresponding primer sets listed in Table 2. The transcription of each gene was normalized to transcript level of the reference gene, the 16S rRNA gene, and used as a housekeeping gene.

### 4.7. Statistical Analysis

Data are presented as the mean ± standard deviation (SD) of at least three independent biological replicates unless otherwise stated. Statistical analyses were performed using one-way analysis of variance (ANOVA) followed by Tukey’s multiple-comparison test. Differences were considered statistically significant at *p* < 0.05. Statistical analyses were conducted using Python 3.14.4.

## Figures and Tables

**Table 1:** Bacterial strains used in the present study.

Species	Strain	Cultivation Temperature	Cultivation Medium	Anaerobicity	Isolation Source
*Enterococcus* sp.	no. 250	37 °C	MRS	Facultative	Longan fruits
*E. faecalis*	no. 372	37 °C	MRS	Facultative	Spinach
*L. pseudomesenteroides*	no. 403	28 °C	MRS	Facultative	Banana
*L. plantarum*	no. 665	28 °C	MRS	Facultative	*Broussonetia* × *kazinoki*
*L. plantarum*	no. 722	37 °C	MRS	Facultative	*Broussonetia* × *kazinoki*
*A. actinomycetemcomitans*	ATCC 29523	37 °C	Modified GAM	Obligate	Blood
*F. nucleatum*	ATCC 25586	37 °C	Modified GAM	Obligate	Cervico-facial lesion
*P. gingivalis*	W83	37 °C	Modified GAM	Obligate	Clinical specimen

**Table 2:** Primes used for qPCR analyses in the present study.

Species	Target		Sequence (5′ → 3′)
*A. actinomycetemcomitans*	16S rRNA	F:	ACGCTGTAAACGGTGTCG
		R:	TTGCATCGAATTAAACCACAT
	*apiA*	F:	GGAAGCTGATCGACTGCTTT
		R:	CCTTCTTGGTGATGGTGATG
	*cdtB*	F:	CAACAACACAATTCCAACCC
		R:	GGCGATACCTGTCCATTCTT
	*dspB*	F:	ATACCATCAGCCTTTCCGGC
		R:	GGCATTTTCCGCACGTTGAT
	*flp*	F:	ATGACCGACGCTGATGTTTA
		R:	TTCGACGGTGATGTTGATGA
	*ltxA*	F:	ATCAGCCCTTTGTCTTTCCTAG
		R:	TGACCAAGTAAACTATCGCCG
	*luxS*	F:	ATGGTGCTGACGTTGATGAA
		R:	CTGTAGCTGCCGTTACTTGA
*F. nucleatum*	16S rRNA	F:	AAGCGCGTCTAGGTGGTTATGT
		R:	TGTAGTTCCGCTTACCTCTCCAG
	*fadA*	F:	GAAGAAAGAGCACAAGCTGA
		R:	GCTTGAAGTCTTTGAGCTCT
	*fap2*	F:	GCTGCTGAAAGCATGGTAGA
		R:	AACTCGTCCAGCCTTCTTCA
	*luxS*	F:	GCAACGGTATCAAGGACTGA
		R:	TCCAGCTTCTTCTTGGTTGA
	*radD*	F:	ATCGACGAGGTTGTTGGTTA
		R:	TTCGACCTGATCGTCAACAG
*P. gingivalis*	16S rRNA	F:	TGTAGATGACTGATGGTGAAA
		R:	ACTGTTAGCAACTACCGATGT
	*fimA*	F:	GCGACGCTATATGCAAGACAAT
		R:	TTACCAAGTAGCAGCCTGATTAA
	*hagA*	F:	TAAATAAGGGCGGAGCAAGA
		R:	GACGGAAAGCAACATACTTCG
	*hem*	F:	ACGAAGCCTTGTTCTCCTCA
		R:	CAATGAATATGCCGGTTTCC
	*luxS*	F:	ATGGCAGCTTTGACGGTATT
		R:	GCTTCTTGGCGTATCAATCC
	*mfa1*	F:	ATGGTGGTGCTGATGCTGAT
		R:	TTCGACCTTCTTGGCATTTG
	*ragA*	F:	CGCTATTCTTCCTTTGCTTGCT
		R:	GATCGTGGTGTTTCCGACAA
	*rgpB*	F:	GCTCGGTCAGGCTCTTTGTA
		R:	GGGTAAGCAGATTGGCGATT

## Data Availability

All data are reported in the manuscript.

## Abbreviations

The following abbreviations are used in this manuscript:

Aa	*A. actinomycetemcomitans*
AI-2	Autoinducer-2
Fn	*F. nucleatum*
GAM	Gifu Anaerobic Medium
HPLC	High-performance liquid chromatography
LAB	Lactic acid bacterium
MRS	de Man, Rogosa, and Sharpe
PBS	Phosphate-buffered saline
Pg	*P. gingivalis*
qRT-PCR	Quantitative reverse transcription–PCR
